# Health Care Resource Utilization and Costs among Adult Patients with Advanced Soft Tissue Sarcoma: A Retrospective Medical Record Review in the United Kingdom, Spain, Germany, and France

**DOI:** 10.1155/2018/2020591

**Published:** 2018-09-12

**Authors:** Daniel S. Mytelka, Saurabh P. Nagar, Yulia D'yachkova, Elizabeth M. La, James A. Kaye, Sean D. Candrilli, Bernd Kasper, Jose Antonio Lopez-Martin, Maria Lorenzo

**Affiliations:** ^1^Eli Lilly and Company, Lilly Corporate Center, Indianapolis, IN 46285, USA; ^2^RTI Health Solutions, 200 Park Offices Drive, Research Triangle Park, NC 27709, USA; ^3^Eli Lilly Gesellschaft m.b.H., Koelblgasse 8-10, 1030 Vienna, Austria; ^4^RTI Health Solutions, 307 Waverley Oaks Road, Suite 101, Waltham, MA 02452, USA; ^5^Mannheim University Medical Center, Theodor-Kutzer-Ufer 1-3, 68167 Mannheim, Germany; ^6^12 de Octubre University Hospital, Avenida de Córdoba, s/n, 28041 Madrid, Spain; ^7^Eli Lilly and Company Limited, Lilly Research Centre, Erl Wood Manor, Sunninghill Road, Windlesham, Surrey GU20 6PH, UK

## Abstract

**Objective:**

To describe health care resource utilization and costs for patients with advanced soft tissue sarcoma (STS) in the United Kingdom (UK), Spain, Germany, and France.

**Methods:**

Physicians abstracted data for adult patients with a diagnosis of advanced STS (other than Kaposi's sarcoma or gastrointestinal stromal tumor) who received ≥1 lines of systemic therapy. Health care resource utilization related to advanced STS treatment was recorded; associated costs were estimated by applying unit costs.

**Results:**

A total of 130 physicians provided data for 807 patients (UK: 199; Spain: 203; Germany: 204; and France: 201). The site of care during active treatment varied based on differences in the health care systems of these four countries. Total mean per-patient health care cost in the UK was £19,457; in Spain, €26,814; in Germany, €20,468; and in France, €24,368. Advanced STS-related systemic treatment costs were driven primarily by drug acquisition and administration costs. Treatment-related costs increased during later lines of therapy for all countries except France, where they decreased after first-line therapy. Pain control and antiemetics were the most common supportive care medications.

**Conclusions:**

This study provides real-world data on resource utilization and estimated costs in advanced STS and could inform policymakers about treatment burden.

## 1. Introduction

There are more than 50 histological subtypes of soft tissue sarcoma (STS), a rare and heterogeneous group of malignant neoplasms [1]. STSs account for approximately 1% of all incident malignancies [2], with an estimated 23,574 incident cases in the European Union in 2013 [3]. An estimated 40% to 50% of patients with STS are initially diagnosed with or later develop metastatic disease [4], for which treatment options are limited. Chemotherapy (e.g., doxorubicin or ifosfamide, alone or in combination with each other or other agents) is most commonly used to treat patients with inoperable, advanced STS [5–8]. The intent of these treatments is primarily palliative, and the response rates are low (i.e., 10%–25% with monotherapy) [6]. Median overall survival among patients with metastatic STS varies depending on histologic subtype, grade, performance status, and other prognostic factors but generally is estimated to be 12 to 18 months [9].

Limited data have been published to date on health care resource use and costs associated with treatment of a broad sample of patients with advanced STS in European countries, despite the considerable disease burden of STS. The Sarcoma Treatment and Burden of Illness in North America and Europe (SABINE) study, a multicenter, multicountry, retrospective medical record review study of a highly restricted population of patients with metastatic STS who demonstrated a favorable response to chemotherapy after four cycles, found that advanced STS is associated with considerable economic burden, and significant resources are devoted to treating it across European countries [10]. The SABINE study [10] focused on lifetime health care resource use (only number of resource use days), estimated costs among STS patients, and presented the results by treatment line, chemotherapy use, and disease progression. Judson et al. [11] appear to be the first researchers in the public domain who provided detailed cost estimates for metastatic STS in the UK. Guest et al. [12] and Amdahl et al. [13] conducted cost-effectiveness analyses (of doxorubicin/ifosfamide versus trabectedin and of pazopanib, resp.). Both reported that use of trabectedin or pazopanib in later lines of therapies can be cost effective.

The objective of this study was to fill gaps in the literature, including providing data on health care resource use (proportion with resource use events and number of visits) and estimating STS-related treatment costs (i.e., drug acquisition, drug administration, and treatment-related adverse events) for patients with advanced STS in real-world clinical settings in the United Kingdom (UK), Spain, Germany, and France. We also evaluated treatment patterns and outcomes in this population; these results are reported separately [14–18].

## 2. Methods

### 2.1. Study Design and Population

This study was a retrospective review of medical records of patients with advanced STS (excluding those diagnosed with Kaposi's sarcoma or gastrointestinal stromal tumor) in the UK, Spain, Germany, and France. Oncologists who had personally treated at least 3 patients with advanced STS in the previous year abstracted anonymized data from their patients' medical records. To enhance geographic representativeness, physicians across all regions of each country were contacted to participate in the study. The dates of chart abstraction in the UK were 7 August 2015 through 5 October 2015; in Germany, 20 August 2015 through 9 November 2015; in Spain, 21 August 2015 through 15 January 2016; and in France, 15 February 2016 through 4 May 2016.

Patients were eligible if diagnosed with advanced, histologically confirmed STS (at presentation or after progression from limited disease), between 1 January 2005 and 18 months before the initiation of record abstraction. Eligible patients were aged 18 years or older at initial diagnosis of STS, had started at least one line of systemic therapy for advanced STS, and had records from diagnosis of advanced STS until the date of record abstraction or death that were accessible to the abstracting physician. Patients were excluded if they had received treatment with curative intent after diagnosis of advanced disease; had received treatment with doxorubicin or other anthracyclines or anthracenediones at any time before initiating first-line treatment for advanced STS; had evidence of concurrent malignancy, except adequately treated nonmelanoma skin cancer or in situ neoplasm; or had received first-line treatment within an interventional clinical trial.

The relevant national competent authorities reviewed and approved the study in all four countries, as appropriate. In Germany and Spain, the ethics committee at the study site of the principal investigator in each country reviewed and approved the study, as required by national authorities. This site-level approval applied to all participating sites within each country. Specifically, the study received ethical review board approval from Medizinische Fakultät Mannheim der Universität Heidelberg, Universitätsklinikum Mannheim (reference ID: 2015-824R-MA) in Germany and received ethical review board approval from Unidad Administrativa Ceic Instituto de Investigación Hospital 12 de Octubre (reference ID: 15/169 EPA-OD; LY3012207) in Spain. In addition, the study received national approval from the Commission Nationale de l'informatique et des Libertés (n/Ref: MMS/CWR/AAR1514906) and Le Comité consultatif sur le traitement de l'information en matière de recherche (CCTIRS) (Dossier no. 15.464bis) in France. In the UK, the National Health Service and National Research Authority considered this study a service evaluation and did not require a detailed ethics review. In addition, the study was determined to be exempt from full review by the RTI International Institutional Review Board.

### 2.2. Study Measures

Patient and disease characteristics and treatments that were abstracted from the medical records included race/ethnicity (except in France, where collection of such information is prohibited), age, sex, STS histology, and the number of lines of therapy received. Individual histologic subtypes were grouped into broader categories based on the World Health Organization Classification of Tumours of Soft Tissue and Bone [1]. Histologic categories subsequently analyzed were leiomyosarcoma (smooth muscle tumours), fibroblastic/myofibroblastic sarcoma, liposarcoma (adipocytic tumor), vascular sarcoma, rhabdomyosarcoma (skeletal muscle tumor), synovial sarcoma, and others or not otherwise specified.

Advanced STS-related health care utilization measures were abstracted directly from the patient's medical record according to care setting (e.g., inpatient, physician office) and whether encounters were for chemotherapy administration. Visits were stratified by the treatment line in which they occurred or, as a separate category, the period from the end of last observed treatment line to the end of the patient record or death. The following data were collected: outpatient consult visits (collected in the UK), office visits (collected in Spain, Germany, and France), outpatient hospital visits (collected in Spain, Germany, and France), palliative care visits, outpatient nurse visits (collected in the UK), accident and emergency or emergency department (ED) visits, inpatient hospitalizations (including hospitalizations after a line of therapy), and transfers to long-term or hospice care. Finally, patients' receipt of specific categories of supportive care was collected.

A cost analysis among the four study countries was then conducted using a payer or health system perspective, as was done in the SABINE study [10]. Specifically, costs related to advanced STS were calculated by multiplying the amount of each type of resource used with a corresponding unit cost (Supplementary Tables 1–4). Systemic anticancer therapy costs were calculated in total and are also presented broken down into those for anticancer therapy drug costs, anticancer therapy administration costs, and treatment-related adverse event costs (except for France, for which drug costs were generally included within administration costs). Other direct health care costs were calculated as the sum of all costs associated with outpatient and ED visits, inpatient stays that were not adverse event-related, selected supportive care services, and inpatient long-term care and hospice care. Reported summary statistics for other direct health care costs were calculated in total and are also presented broken down into those for period of follow-up and weekly costs, with measures provided both with and without surgery/radiotherapy. Because cost data are not available in patient medical records, unit costs were obtained for the various products and services from published literature and other standard cost sources. Specifically, systemic anticancer therapy and supportive care costs were obtained for the UK from the British National Formulary [19] and MIMS [20]; for Spain from BOT PLUS [21], and for Germany from Lauer-Taxe [22]. For France, supportive care costs were obtained from Base des Médicaments et Informations Tarifaires [23]. Systemic anticancer drug administration costs were obtained for the UK from the National Health Service [24], for Spain from E-Salud [25], for Germany from Kassenärztliche Vereinigung Nordrhein [26] and Kassenärztliche Vereinigung Sachsen [27], and for France from data on file sources. Unit costs for all four countries were adjusted to 2015/2016 values using relevant inflation indices (in the UK, Curtis and Burns [28]; in Spain, INEbase [29]; in Germany, Statistisches Bundesamt [30]; and in France, INSEE database [31]).

### 2.3. Statistical Analyses

All analyses were descriptive and exploratory in nature and conducted using the SAS software package (version 9.4). No formal statistical tests comparing results across countries or lines of therapy were conducted. The results are presented in tabular displays of frequencies, proportions, means, and other descriptive statistics as appropriate.

Systemic anticancer therapy costs were estimated for each of the five most common first-line, second-line, and third-line regimens identified (with all other regimens in each line reported as a group in an “other” category). Other direct health care costs were estimated by line of treatment, through third line, as well as after discontinuation of first- and second-line treatment.

Health care resource use and costs were summarized only for patients for whom the number of visits/encounters for each particular resource category was available. For instance, a physician may have indicated that a patient was hospitalized, but the data needed to quantify the number of unique hospitalizations or inpatient visits were not recorded; such patients would be included in the count of the number (ever) hospitalized but would not be included in the calculation of the total number of hospitalizations. Annual health care utilization rates were calculated by dividing the number of visits by the observation time (duration of treatment line in years).

## 3. Results

### 3.1. Physician Characteristics

Overall, 130 physicians participated in the study (21 in the UK, 34 in Spain, 40 in Germany, and 35 in France). In the previous year, the participating physicians reported treating an average of 45 patients (standard deviation, 37) with advanced STS.

### 3.2. Patient Characteristics

Overall, 807 patients were included in the study population: 199 in the UK, 203 in Spain, 204 in Germany, and 201 in France (Table 1). The population was 59% male in the overall sample (UK, 63%; Spain, 61%; Germany, 64%; France, 48%), and 93% of patients were white (UK, 90%; Spain, 99%; Germany, 90%; race/ethnicity not collected in France). Mean age at the time of advanced diagnosis was 57.1 years (range, 21–90 years). The median observed follow-up time (from diagnosis of advanced STS to death or the last medical encounter before the abstraction date) was 20.3 months (range, 0–133 months). The most common identified histologic categories were leiomyosarcoma (29%), liposarcoma (13%), and rhabdomyosarcoma (11%). All patients had received at least one line of therapy (as an eligibility criterion), and the mean total number of lines of therapy received was 1.6 (standard deviation, 0.8).

### 3.3. Health Care Resource Utilization

Table 2 presents resource utilization during active treatment with systemic therapy for advanced STS in the UK, Spain, Germany, and France; Supplementary Tables 5–8 present resource utilization by line of therapy for each country.

During active treatment, the patients received substantial amounts of care, although the predominant site of care varied somewhat based on the health care system. Care in the outpatient setting (i.e., outpatient visits to a hospital clinic) was common in all countries, with a mean of 15.0, 26.4, 22.4, and 38.7 outpatient visits per year (annual rate) among patients with at least one visit in France, the UK, Spain, and Germany, respectively. The majority of outpatient visits included chemotherapy administration, although more than 30% of the patients from France had chemotherapy administered in other venues (often office or inpatient visits) (Supplementary Table 12).

Utilization of other sites of care varied by country. In Spain, 55% of patients had outpatient palliative care visits (versus 13%–24% in other countries), and outpatient nurse visits were utilized only in the UK. More patients from Spain had ED visits (86% versus 18%–24% in other countries), while France had both the highest proportion of patients having inpatient visits (46% versus 17%–37% in other countries) and the most annualized inpatient visits per patient with at least one such visit (12 versus 4-5).

End-of-life care rarely included long-term care in any of the study countries (<3%). Hospice care was relatively common in Germany (40%), rarer in Spain and the UK (27% and 19%, resp.), and not received by any patients in France.

The most common supportive care medications (Table 3) were for pain control (UK, 54.8%; Spain, 65.0%; Germany, 60.8%; France, 50.3%) and antiemetics (UK, 44.2%; Spain, 59.6%; Germany, 50.0%; France, 69.7%). Opioid analgesics were the most common form of pain medication (among those who received pain medication: UK, 89.0%; Spain, 84.9%; Germany, 74.2%; France, 83.2%).

### 3.4. Health Care Costs

Tables 4 [Table tab5] [Table tab6]–7 present estimated health care costs related to first-, second-, and third-line treatment of advanced STS by country; detailed costs for the five most common regimens and the other combined regimens are presented in Supplementary Tables 13–16.

Estimated advanced STS-related systemic treatment costs (i.e., drug acquisition, administration, and treatment-related costs) were greater than other estimated direct health care costs in each country, largely driven by drug acquisition and administration costs. Drug acquisition costs increased across lines of therapy in the UK (ranging from £5,998 in the first line to £20,586 in the third line), Spain (€2,029 to €25,446, resp.), and Germany (€4,399 to €23,540, resp.), potentially driven by the more common use of branded agents such as pazopanib (Votrient) and trabectedin (Yondelis) in later lines of therapy than in the first line (see Supplementary Tables 13–15). However, in France, estimated drug acquisition and administration costs were greatest in the first line of therapy (€8,708) and decreased across subsequent lines of therapy, to €6,563 in the third line. The high cost of first-line therapy in France could be driven primarily by greater utilization (number of administrations) of doxorubicin-ifosfamide relative to other countries (Supplementary Table 16) and greater costs for single-agent pazopanib in the first line relative to other lines of therapy (data not presented). Estimated total systemic treatment costs across all lines of therapy (drug acquisition, administration, and treatment-related adverse event costs combined) were £16,363 in the UK, €19,264 in Spain, €16,662 in Germany, and €17,433 in France (Tables 4 [Table tab5] [Table tab6]–7).

Advanced STS-related costs unrelated to anticancer therapy were captured for outpatient visits, accident and emergency/ED visits, inpatient hospitalizations, inpatient long-term care and hospice care, and supportive care. The cost by line of therapy did not change consistently across countries. Overall, estimated mean costs unrelated to anticancer therapy through the end of follow-up or until death were £3,094 in the UK, €7,550 in Spain, €3,806 in Germany, and €6,935 in France (Tables 4 [Table tab5] [Table tab6]–7).

The total mean per-patient health care cost (including systemic treatment-related costs across all lines of therapy and health care costs unrelated to anticancer therapy during follow-up) in the UK was £19,457, in Spain was €26,814, in Germany was €20,468, and in France was €24,368.

## 4. Discussion

This study was a retrospective analysis of medical records of patients with advanced STS who received systemic treatment in the UK, Spain, Germany, and France. The objective of the study was to present a descriptive review of the estimated costs of treatment in these countries. It should be noted that owing to differences across countries in the organization of medical care and other potentially confounding factors, the study was not designed to compare results among the countries, although substantial variations were noted where apparent. In particular, some notable variations in health care resource utilization across the four countries were observed, including more inpatient visits reported in France and greater numbers of outpatient and ED visits reported in Spain compared with the other countries. Variations in the organization of medical care across the study countries may be an explanation for these differences, such as chemotherapy being administered primarily in hospital settings in France [32].

Before this study, there had been little published data on resource use and costs associated with STS. Nonetheless, the findings of previous studies, particularly the SABINE study [10], provide context for this study. Both studies reported that systemic treatment was the greatest cost driver, representing 65% to 80% of total costs in our study (versus about 52% for treatment plus concomitant medications in SABINE), and that costs generally rose in later lines of therapy [10]. However, estimated absolute costs were much higher in the SABINE study. Our study reported mean total cost per patient to be £19,457 in the UK (during a mean follow-up of 23 months), €26,814 in Spain (during a mean follow-up of 27 months), €24,368 in France (during a mean follow-up of 30 months), and €20,468 in Germany (during a mean follow-up of 18 months). In comparison, SABINE reported higher expected per-patient costs of €36,271 in the UK (during an expected follow-up of 29 months), €87,490 in Spain (during an expected follow-up of 47 months), €55,288 in France (during an expected follow-up of 36 months), and €228,661 in Germany (during an expected follow-up of 83 months) [10]. These differences probably relate to the requirement in the SABINE study that patients had a favorable response to at least one line of chemotherapy, which likely influenced both the number of lines of therapy and the length of therapy; 65% of patients received at least three lines of therapy in SABINE, compared with only 11% in our study. For Germany, the SABINE study included only patients alive at the time of chart abstraction.

Judson et al. [11] conducted a retrospective study in the UK and reported direct medical costs of STS to be £12,019 per patient. The population in that study was somewhat similar to our sample in that, on average, patients received 1.6 lines of therapy, compared with 1.4 among UK patients in our study. However, Judson and colleagues' study was conducted before the approval of newer treatments for advanced STS such as pazopanib and trabectedin, which limits comparability of the studies. Although previous cost-effectiveness studies (e.g., Guest et al. [12] and Amdahl et al. [13]) have reported on costs in advanced STS, their results are not directly comparable with ours, owing to differences in the study design and sources used for cost estimation. Guest et al. [12] relied on expert interviews to derive estimates of resource utilization, and Amdahl et al. [13] used resource utilization as reported in a clinical trial.

Given the limited data available on the costs of advanced STS, published data on costs of two other cancers with similar survival might provide additional context for our results. Using a study design and methodology similar to the current study, Kurosky et al. [33] conducted an observational review of medical records in patients with non-small-cell lung cancer (NSCLC) in France, Germany, Spain, and the UK to describe health care resource use and costs associated with the disease [33], and a retrospective study combining data from the National Cancer Data Repository, Hospital Episode Statistics, and the National Schedules of Reference Costs estimated the incidence and prevalence costs during the first year after diagnosis with colorectal cancer in England [34]. To compare these reported costs for NSCLC and colorectal cancer with the current study, we calculated the monthly and annual costs during active treatment of STS by summing drug costs, administration costs, adverse event costs, and costs related to other health care resource utilization across each line of therapy and divided them by the duration of treatment in months for each country. Kurosky and colleagues estimated mean monthly costs of £1,985, €2,394, €3,137, and €4,178 during active treatment of NSCLC in the UK, Spain, Germany, and France, respectively [33], while estimated mean monthly costs (converted to euros for the UK) during active treatment of STS were €1,671, €1,518, €1,430, and €1,440 in the UK, Spain, Germany, and France, respectively. Laudicella et al. [34] reported average total costs per colorectal cancer patient (aged 18–64 years) of £17,241 compared with £9,528 during the first year after diagnosis with advanced STS in our study (data not presented). Costs related to NSCLC and colorectal cancer were higher than costs reported for advanced STS, which could reflect the slower progress toward the development of effective treatment options for STS.

Previous research has identified considerable symptom burden in STS, with pain being among the most prevalent symptoms [35]. Kuo et al. [36] conducted a study of an STS population in a UK sarcoma unit and found that more than half of assessed patients reported pain. Gough et al. [37] conducted a medical record abstraction in the UK and deduced that patients with metastatic STS have a significant symptom burden, with pain being a substantial problem. Our results indicate that more than half of the patients in each country utilized some form of pain medication, with more than 74% of those in each country reported opioid use to control pain. Our results are consistent with previous research and support the need to focus on early palliative treatment.

The results of this study are subject to several limitations inherent to many retrospective medical record review studies. Although we attempted to achieve balance in geographic distribution within and across countries, the participating physicians and patient sample may not be completely representative of the population in the countries studied. The extent to which physicians self-selected for participation in this study is unknown and could influence results. Because of the patient inclusion and exclusion criteria (including previous treatments rendering a patient ineligible for the study), the sample is subject to selection bias, and the study findings may not be fully generalizable to the entire population of adult patients with advanced STS who undergo systemic treatment in the study countries. Moreover, due to administrative censoring, it is possible that the number of lines of therapy could be underestimated. Physicians reported data based on information available in the patients' medical records to which they had access; although major events such as inpatient hospitalizations are likely to have been recorded, it is possible that patients could have received health care services in other care settings that were not reported back to the treating physician and therefore were not part of their medical record and would not be captured in this study. Finally, the practice patterns may have changed from 2005, particularly with the introduction of new treatments and guidelines that were not available for patients treated in earlier years; date of advanced STS diagnosis was not collected, and therefore an analysis of time trends in treatment was not possible.

## 5. Conclusions

This was one of the first large-scale, real-world studies of health care utilization and estimated associated costs in a multinational population of patients with advanced STS who received systemic treatment. Treatment costs were substantial in this population, primarily relating to systemic therapy but were less than costs for other cancers such as non-small-cell lung cancer and colorectal cancer, where patients have similar expected survival. Pain is a very common issue in patients with STS, often treated by opiate analgesics.

This study, together with findings from other economic analyses, may help inform future economic and health technology assessments for novel treatments for advanced STS.

## Figures and Tables

**Table 1:** Patient and treatment characteristics.

	Overall		United Kingdom		Spain		Germany		France	
	*n*	(%)	*n*	(%)	*n*	(%)	*n*	(%)	*n*	(%)
Total patients	807	100	199	100	203	100	204	100	201	100
Sex										
** **Male	475	58.9	126	63.3	123	60.6	130	63.7	96	47.8
** **Female	332	41.1	73	36.7	80	39.4	74	36.3	105	52.2
Ethnicity^a^										
** **White/Caucasian	562	92.7	178	89.5	201	99.0	183	89.7	—	—
** **African/black	14	2.3	10	5.0	0	0	4	2.0	—	—
** **Asian or Pacific Islander	14	2.3	6	3.0	0	0	8	3.9	—	—
** **Middle Eastern	8	1.3	2	1.0	2	1.0	4	2.0	—	—
** **Indian subcontinent	8	1.3	3	1.5	0	0	5	2.5	—	—
Age at advanced diagnosis										
** **Mean (SD)	57.1	12.3	56.5	12.4	56.6	13.1	57.4	11.0	57.9	12.6
** **Range (min, max)	21	90	21	90	21	85	28	77	22	84
Histological category										
** **Leiomyosarcoma	229	28.4	61	30.7	45	22.2	66	32.4	57	28.4
** **Others/NOS	183	22.7	40	20.1	35	17.2	43	21.1	65	32.3
** **Fibroblastic/myofibroblastic	78	9.7	24	12.1	18	8.9	20	9.8	16	8.0
** **Liposarcoma	105	13.0	14	7.0	44	21.7	22	10.8	25	12.4
** **Vascular sarcoma	79	9.8	16	8.0	25	12.3	17	8.3	21	10.4
** **Rhabdomyosarcoma	86	10.7	33	16.6	20	9.9	21	10.3	12	6.0
** **Synovial sarcoma	47	5.8	11	5.5	16	7.9	15	7.4	5	2.5
Total number of lines of therapy received										
** **Mean (SD)	1.6	0.8	1.4	0.6	1.6	0.8	1.47	0.6	1.9	1.0
** **Distribution										
** **1	456	56.5	124	62.3	117	57.6	122	59.8	93	46.3
** **2	262	32.5	64	32.2	66	32.5	70	34.3	62	30.9
** **3+	89	11.0	11	5.5	20	9.9	12	5.9	46	22.9

NOS = not otherwise specified; SD = standard deviation. ^a^Not collected in France.

**Table 2:** Health care resource utilization from diagnosis of advanced soft tissue sarcoma, by country.

	United Kingdom		Spain		Germany		France	
	*n*	(%)	*n*	(%)	*n*	(%)	*n*	(%)
Total patients	199	100	203	100	204	100	201	100
*Outpatient visits in a hospital clinic*								
Known number of outpatient visits, *N* (%)	165	82.9	132	65.0	186	91.2	151	75.1
** **At least 1 outpatient visit, *N* (%)	164	99.4	127	96.2	176	94.6	105	69.5
** **Annual number of visits (among patients with at least 1 visit)								
Mean (SD)	26.4	48.4	22.4	14.5	38.7	27.2	15.0	19.9
Median	19.8		19.5		33.7		11.2	
Known number of outpatient chemotherapy administrations, *N* (%)^a^	164	99.4	120	90.9	176	94.6	96	63.6
** **At least 1 outpatient chemotherapy administration, *N* (%)^b^	162	98.8	115	90.6	174	98.9	85	81.0
** **Annual number of outpatient visits for chemotherapy administration (among patients with at least 1 chemotherapy-related visit)								
Mean (SD)	18.1	23.8	16.8	9.7	30.2	21.2	15.2	20.5
Median	15.5		14.6		26.2		11.6	
*Outpatient palliative care visits*								
Known number of outpatient palliative care visits, *N* (%)	104	52.3	128	63.1	52	25.5	147	73.1
** **At least 1 outpatient palliative care visit, *N* (%)	25	24.0	70	54.7	7	13.5	27	18.4
** **Annual number of visits (among patients with at least 1 visit)								
Mean (SD)	5.2	6.0	6.2	5.0	2.7	1.4	5.5	5.7
Median	4.0		4.7		3.0		3.6	
Known number of chemotherapy administrations, *N* (%)^a^	25	24.0	68	53.1	3	5.8	27	18.4
** **At least 1 chemotherapy administration, *N* (%)^b^	6	24.0	4	5.9	0	0	4	14.8
** **Annual number of visits for chemotherapy administration (among patients with at least 1 chemotherapy-related visit)								
Mean (SD)	7.0	6.8	2.9	1			5.0	4.7
Median	4.4		2.8				3.9	
*Outpatient nurse visits*								
Known number of outpatient nurse visits, *N* (%)	96	48.2	0	0	0	0	0	0
** **At least 1 outpatient nurse visit, *N* (%)	47	49.0	0		0		0	
** **Annual number of visits (among patients with at least 1 visit)								
Mean (SD)	5.7	3.6						
Median	5.0							
Known number of chemotherapy administrations, *N* (%)^a^	47	49.0						
** **At least one chemotherapy administration, *N* (%)^b^	24	51.1	0	0	0	0	0	0
** **Number of visits for chemotherapy administration (among patients with at least 1 chemotherapy-related visit)			0		0		0	
Mean (SD)	6.8	3.7						
Median	6.1							
*A&E/ED visit* ^c^								
Known number of A&E/ED visits, *N* (%)	110	55.3	132	65.0	46	22.6	144	71.6
** **At least 1 A&E/ED visit, *N* (%)	20	18.2	113	85.6	9	19.6	34	23.6
** **Annual number of visits (among patients with at least 1 visit)								
Mean (SD)	3.2	2.0	6.4	17.1	10.3	16.0	16.0	61.6
Median	2.7		3.7		4.9		2.7	
Known number of chemotherapy administrations, *N* (%)^a^	20	18.2	108	81.8	8	17.4	30	20.8
** **At least 1 chemotherapy administration, *N* (%)^b^	7	35.0	31	27.4	1	11.1	8	23.5
** **Annual number of visits for chemotherapy administration (among patients with at least 1 chemotherapy-related visit)								
Mean (SD)	3.2	2.4	2.5	2.3	38.6		51.0	125.1
Median	2.6		1.8		38.6		4.73	
*Inpatient visits*								
At least 1 inpatient visit, *N* (%)	34	17.1	75	36.9	45	22.1	92	45.8
** **Annual number of visits (among patients with at least 1 visit)								
Mean (SD)	4.1	4.85	5.2	20.6	5.0	5.1	12.4	11.3
Median	2.6		2.2		3.3		9.2	
*Office visit*	—	—						
Known number of office visits, *N* (%)	—	—	115	56.7	60	29.4	159	79.1
** **At least 1 office visit, *N* (%)	—	—	49	42.6	28	46.7	122	76.7
** **Annual number of visits (among patients with at least 1 visit)	—	—						
Mean (SD)	—	—	9.4	11.6	32.9	23.2	19.9	66.4
Median	—	—	5.4		26.8		9.3	
Known number of chemotherapy administrations, *N* (%)^a^	—	—	42	36.5	27	45.0	111	69.8
** **At least 1 chemotherapy administration, *N* (%)^b^	—	—	20	40.8	26	92.9	71	58.2
** **Annual number of visits for chemotherapy administration (among patients with at least 1 chemotherapy-related visit)	—	—						
Mean (SD)	—	—	9.0	8.1	29.3	25.0	11.3	7.2
Median	—	—	7.5		19.7		11.2	
*Received long-term care, N (%)*	2	1.0	6	3.0	1	0.5	4	2.0
*Received hospice care, N (%)*	38	19.1	55	27.1	81	39.7	0	0

A&E = accident and emergency; ED = emergency department; SD = standard deviation. ^a^Percentage of patients with a known number of visits. ^b^Percentage of patients with at least one visit. Note: for this calculation, patients without a known number of chemotherapy administrations were assumed to have no chemotherapy administrations. ^c^Accident and emergency visits were collected in the UK; emergency department visits were collected in Spain, Germany, and France.

**Table 3:** Use of supportive/palliative medications from time of advanced STS diagnosis to death.

	United Kingdom		Spain		Germany		France	
	*n*	(%)	*n*	(%)	*n*	(%)	*n*	(%)
Total patients	199	100	203	100	204	100	201	100
Supportive/palliative medication use								
** **Pain control	109	54.8	132	65.0	124	60.8	101	50.3
** **Antiemetics	88	44.2	121	59.6	102	50	140	69.7
** **Antibiotics	22	11.1	53	26.1	32	15.7	30	14.9
** **Antifungals	8	4.0	8	3.9	11	5.4	11	5.5
** **Antivirals	0	0	5	2.5	2	1.0	0	0
** **Dexrazoxane	0	0	0	0	0	0	3	1.5
** **Transfusions	20	10.1	32	15.8	24	11.8	30	14.9
** **Antianxiety	10	5.0	45	22.2	18	8.8	37	18.4
** **Growth factors	15	7.5	45	22.2	19	9.3	93	46.3
** **Erythropoiesis-stimulating agents	0	0.0	8	17.8	0	0.0	23	24.7
** **Bisphosphonates	1	0.5	5	2.5	4	2.0	12	6.0
** **Referral to psychiatrist, psychologist, or other professionals for distress management	7	3.5	23	11.3	14	6.9	19	9.5
** **Oxygen	11	5.5	23	11.3	17	8.3	17	8.5
** **Radiation therapy, excluding utilization for pain control	4	2.0	16	7.9	3	1.5	7	3.5
** **Nutritional support	30	15.1	50	24.6	37	18.1	43	21.4
** **Corticosteroids, except for utilization for pain control	25	12.6	62	30.5	38	18.6	82	40.8
** **Patient did not receive any supportive care	28	14.1	14	6.9	45	22.1	42	20.9
** **Others	1	0.5	1	0.5	0	0	1	0.5
** **Unknown	39	19.6	17	8.4	16	7.8	13	6.5
Treatment for pain control among those who received pain control medications	109	100.0	132	100.0	124	100.0	101	100.0
** **Opiate analgesics	97	89.0	112	84.9	92	74.2	84	83.2
** **Other analgesics	59	54.1	74	56.1	74	59.7	47	46.5
** **Radiotherapy	13	11.9	30	22.7	10	8.1	8	7.9
** **Psychological intervention	5	4.6	14	10.6	2	1.6	13	12.9
** **Sedation for refractory pain	3	2.8	12	9.1	0	0	10	9.9
** **Epidural infusions	0	0	2	1.5	0	0	1	1.0
** **Splanchnic nerve block	0	0	0	0	0	0	1	1.0
** **Others	1	0.9	0	0	0	0	0	0
** **Unknown	0	0	0	0	0	0	2	2.0

**Table 4:** Estimated health care costs related to treatment of advanced STS^a^ in the United Kingdom (*n*=199).

	First line^d^	Second line^d^	Third line^d^
*Systemic treatment costs*			
*N* (%)	199 (100)	75 (100)	11 (100)
Drug costs			
** **Mean (SD)	£5,997.70 (£8,292.80)	£10,850.80 (£10,842.50)	£20,586.40 (£21,772.90)
** **Median	£2,954.90	£7,352.30	£11,623.00
** **Range (min, max)	(£247.80, £65,646.00)	(£259.90, £50,849.90)	(£3,121.90, £78,422.30)
Administration costs^b^			
** **Mean (SD)	£3,200.60 (£2,145.90)	£3,122.30 (£1,482.30)	£3,311.00 (£2,154.70)
** **Median	£2,029.10	£3,042.70	£2,756.50
** **Range (min, max)	(£608.60, £18,054.40)	(£338.20, £8,479.50)	(£1,366.10, £9,093.20)
Treatment-related adverse event costs^c^			
** **Mean (SD)	£271.90 (£1,055.90)	£526.20 (£1,696.20)	£754.70 (£2,503.20)
** **Median	£0.00	£0.00	£0.00
** **Range (min, max)	(£0.00, £9,503.20)	(£0.00, £8,386.50)	(£0.00, £8,302.10)
Total (all lines of therapy combined)			
** **Mean (SD)	£16,362.80 (£15,474.90)		
** **Median	£12,854.98		
** **Range (min, max)	(£1,349.28, £103,887.69)		
*Other direct health care costs* ^e^			
*N* (%)	199 (100)	75 (100)	11 (100)
Total cost			
** **Mean (SD)	£2,064.70 (£4,001.30)	£1,357.90 (£3,296.30)	£3,331.40 (£5,830.90)
** **Median	£658.70	£308.30	£454.10
** **Range (min, max)	(£0.00, £26,323.90)	(£0.00, £23,835.20)	(£0.00, £19,218.50)
Total cost (all available follow-ups)			
** **Mean (SD)	£3,093.84 (£5,193.07)		
** **Median	£1,038.76		
** **Range (min, max)	(£0.00, £32,239.68)		
Total cost, excluding surgery and radiotherapy			
** **Mean (SD)	£1,928.40 (£3,720.30)	£1,217.40 (£3,233.90)	£3,091.90 (£5,783.20)
** **Median	£637.90	£253.20	£454.10
** **Range (min, max)	(£0.00, £26,323.90)	(£0.00, £23,835.20)	(£0.00, £19,218.50)
Period of follow-up (weeks)			
** **Mean (SD)	24.20 (20.30)	22.60 (13.00)	21.60 (17.00)
** **Median	20.10	21.10	18.00
** **Range (min, max)	(0.10, 177.30)	(0.10, 68.30)	(1.90, 68.10)
Weekly cost			
** **Mean (SD)	£130.80 (£341.90)	£70.50 (£147.80)	£1,011.70 (£3,099.40)
** **Median	£36.20	£16.90	£17.50
** **Range (min, max)	(£0.00, £2,897.10)	(£0.00, £817.90)	(£0.00, £10,348.40)
Weekly cost, excluding surgery and radiotherapy			
** **Mean (SD)	£121.80 (£319.70)	£63.50 (£143.50)	£1,000.80 (£3,102.60)
** **Median	£34.30	£12.90	£17.50
** **Range (min, max)	(£0.00, £2,751.80)	(£0.00, £817.90)	(£0.00, £10,348.40)

SD = standard deviation. Note: systemic treatment administration costs include the costs of administration and the costs of outpatient visits or hospitalizations that were for chemotherapy administration. Treatment-related adverse event costs include costs associated with growth factors (which were assumed to have been used to prevent and/or treat neutropenia or anemia) and costs associated with hospitalizations to manage toxicities/side effects related to treatments or procedures. For systemic treatment costs by line of therapy, costs are reported among those patients who received each line of therapy. Costs for all patients are reported by line of therapy, but costs for those using specific treatment regimens are not provided for third- and fourth-line patients due to small sample sizes. ^a^Costs are in 2015/2016 British pounds and were inflated when necessary using the Hospital and Community Health Services price inflation index [27]. ^b^Systemic treatment administration costs include the costs of administration, as well as the costs of outpatient visits or hospitalizations that were for chemotherapy administration. ^c^Treatment-related adverse event costs include costs associated with growth factors (which were assumed to have been used to prevent and/or treat neutropenia or anemia), as well as costs associated with hospitalizations to manage toxicities/side effects related to treatments or procedures. ^d^Costs are reported among those patients who received each line of therapy. ^e^Other direct health care costs include costs unrelated to anticancer therapy (i.e., other direct health care costs exclude costs associated with anticancer drug acquisition and administration and treatment-related adverse events). These other direct health care costs include costs associated with outpatient visits, accident and emergency visits, inpatient hospitalizations, inpatient long-term care and hospice care, and supportive care.

**Table 5 tab5:** Estimated health care costs related to treatment of advanced STS^a^ in Spain (*n*=203).

	First line^d^	Second line^d^	Third line^d^
*Systemic treatment costs*			
*N* (%)^b^	202 (100)	86 (100)	20 (100)
Drug costs			
** **Mean (SD)	€2,028.60 (€5,280.70)	€9,862.90 (€18,056.20)	€25,445.50 (€25,825.00)
** **Median	€543.20	€1,924.60	€20,132.90
** **Range (min, max)	(€36.10, €47,880.00)	(€65.10, €128,404.60)	(€59.80, €84,993.90)
Administration costs^c^			
** **Mean (SD)	€5,599.50 (€5,875.20)	€5,406.10 (€4,818.90)	€4,088.90 (€2,362.80)
** **Median	€4,030.90	€3,439.20	€4,022.20
** **Range (min, max)	(0.00, €37,900.10)	(0.00, €24,671.00)	(€0.00, €8,412.20)
Treatment-related adverse event costs^e^			
** **Mean (SD)	€1,259.80 (€3,442.20)	€1,087.00 (€3,058.10)	€1,505.80 (€3,371.60)
** **Median	€0.00	€0.00	€0.00
** **Range (min, max)	(€0.00, €21,611.80)	(€0.00, €20,840.00)	(€0.00, €11,247.50)
Total (all lines of therapy combined)			
** **Mean (SD)	€19,263.87 (€30,628.10)		
** **Median	€12,854.98		
** **Range (min, max)	(€185.38, €167,622.11)		
*Other direct health care costs* ^f^			
*N* (%)	202 (100)	86 (100)	20 (100)
Total cost			
** **Mean (SD)	€3,795.10 (€6,457.20)	€5,244.60 (€7,340.10)	€8,588.00 (€12,794.80)
** **Median	€1,507.40	€2,168.40	€2,671.50
** **Range (min, max)	(€0.00, €46,483.30)	(€0.70, €32,436.90)	(€130.00, €40,032.70)
Total cost (all available follow-ups)			
** **Mean (SD)	€7,549.84 (€10,131.67)		
** **Median	€3,793.44		
** **Range (min, max)	(€0.00, €51,666.24)		
Total cost, excluding surgery and radiotherapy			
** **Mean (SD)	€3,640.10 (€6,310.00)	€5,146.20 (€7,333.90)	€6,823.50 (€11,107.20)
** **Median	€1,466.50	€2,051.80	€2,044.20
** **Range (min, max)	(€0.00, €46,483.30)	(€0.00, €32,436.90)	(€130.00, €40,032.70)
Period of follow-up (weeks)			
** **Mean (SD)	24.60 (14.50)	23.00 (14.60)	25.30 (17.80)
** **Median	22.10	21.20	19.20
** **Range (min, max)	(0.40, 95.70)	(0.10, 78.10)	(6.10, 64.10)
Weekly cost			
** **Mean (SD)	€230.80 (€627.10)	€354.40 (€660.40)	€296.70 (€343.60)
** **Median	€76.40	€153.80	€110.00
** **Range (min, max)	(€0.00, €7,191.40)	(€0.20, €4,535.90)	(€21.20, €1,376.60)
Weekly cost, excluding surgery and radiotherapy			
** **Mean (SD)	€224.90 (€625.70)	€296.00 (€477.00)	€229.60 (€234.80)
** **Median	€76.00	€133.00	€103.60
** **Range (min, max)	(€0.00, €7,191.40)	(€0.00, €2,428.60)	(€21.20, €697.10)

SD = standard deviation. Note: systemic treatment administration costs include the costs of administration and the costs of outpatient visits or hospitalizations that were for chemotherapy administration. Treatment-related adverse event costs include costs associated with growth factors (which were assumed to have been used to prevent and/or treat neutropenia or anemia) and costs associated with hospitalizations to manage toxicities/side effects related to treatments or procedures. For systemic treatment costs by line of therapy, costs are reported among those patients who received each line of therapy. Costs for all patients are reported by line of therapy, but costs for those using specific treatment regimens are not provided for third- and fourth-line patients due to small sample sizes. ^a^Costs are in 2016 euros and were inflated when necessary using the indice de precios de consumo, medicina [28]. ^b^One patient with dactinomycin use was excluded from this analysis. ^c^Systemic treatment administration costs include the costs of administration, as well as the costs of outpatient visits or hospitalizations that were for chemotherapy administration. ^d^Costs are reported among those patients who received each line of therapy. ^e^Treatment-related adverse event costs include costs associated with growth factors (which were assumed to have been used to prevent and/or treat neutropenia or anemia), as well as costs associated with hospitalizations to manage toxicities/side effects related to treatments or procedures. ^f^Other direct health care costs include costs unrelated to anticancer therapy (i.e., other direct health care costs exclude costs associated with anticancer drug acquisition and administration and treatment-related adverse events). These other direct health care costs include costs associated with outpatient visits, accident and emergency visits, inpatient hospitalizations, inpatient long-term care and hospice care, and supportive care.

**Table 6 tab6:** Estimated health care costs related to treatment of advanced STS^a^ in Germany (*n*=204).

	First line^d^	Second line^d^	Third line^d^
*Systemic treatment costs*			
*N* (%)	204 (100)	82 (100)	12 (100)
Drug costs			
** **Mean (SD)	€4,399.10 (€8,671.50)	€14,063.70 (€21,170.80)	€23,539.60 (€19,434.40)
** **Median	€1,779.30	€3,469.20	€21,611.00
** **Range (min, max)	(€272.80, €68,732.20)	(€319.40, €91,996.20)	(€429.70, €64,436.40)
Administration costs^b^			
** **Mean (SD)	€1,901.30 (€664.80)	€1,813.30 (€882.40)	€1,195.40 (€630.40)
** **Median	€2,001.60	€1,913.40	€1,169.60
** **Range (min, max)	(€333.60, €3,837.20)	(€309.80, €3,760.50)	(€309.80, €2,001.60)
Treatment-related adverse event costs^c^			
** **Mean (SD)	€1,709.40 (€5,866.30)	€1,997.90 (€10,142.10)	€200.10 (€693.00)
** **Median	€0.00	€0.00	€0.00
** **Range (min, max)	(€0.00, €34,164.90)	(€0.00, €81,727.80)	(€0.00, €2,400.60)
Total (all lines of therapy combined)			
** **Mean (SD)	€16,661.58 (€22,712.93)		
** **Median	€7,756.63		
** **Range (min, max)	(€606.38, €180,829.28)		
*Other direct health care costs* ^e^			
*N* (%)	204 (100)	82 (100)	12 (100)
Total cost			
** **Mean (SD)	€2,229.10 (€6,064.40)	€2,671.00 (€10,330.50)	€1,017.10 (€1,220.60)
** **Median	€569.20	€575.50	€806.30
** **Range (min, max)	(€57.10, €35,665.80)	(€0.00, €83,826.40)	(€110.40, €3,801.60)
Total cost (all available follow-ups)			
** **Mean (SD)	€3,806.37 (€8,995.35)		
** **Median	€1,282.40		
** **Range (min, max)	(€57.10, €84,290.97)		
Total cost, excluding surgery and radiotherapy			
** **Mean (SD)	€2,215.60 (€6,055.20)	€2,663.20 (€10,330.80)	€981.70 (€1,180.00)
** **Median	€566.80	€557.20	€748.60
** **Range (min, max)	(€57.10, €35,665.80)	(€0.00, €83,826.40)	(€110.40, €3,589.40)
Period of follow-up (weeks)			
** **Mean (SD)	17.80 (9.20)	21.30 (16.10)	17.50 (17.70)
** **Median	16.30	18.40	12.00
** **Range (min, max)	(0.10, 69.10)	(2.00, 87.70)	(0.10, 66.00)
Weekly cost			
** **Mean (SD)	€273.40 (€1,183.50)	€114.40 (€310.90)	€132.40 (€226.40)
** **Median	€35.60	€35.90	€46.60
** **Range (min, max)	€2.70 (€14,975.10)	€0.00 (€2,010.10)	€6.90 (€773.10)
Weekly cost, excluding surgery and radiotherapy			
** **Mean (SD)	€263.70 (€1,161.60)	€112.90 (€308.30)	€129.30 (€224.80)
** **Median	€35.30	€35.90	€46.60
** **Range (min, max)	(€2.70, €14,975.10)	(€0.00, €2,010.10)	(€6.90, €773.10)

SD = standard deviation. Note: Systemic treatment administration costs include the costs of administration and the costs of outpatient visits or hospitalizations that were for chemotherapy administration. Treatment-related adverse event costs include costs associated with growth factors (which were assumed to have been used to prevent and/or treat neutropenia or anemia) and costs associated with hospitalizations to manage toxicities/side effects related to treatments or procedures. For systemic treatment costs by line of therapy, costs are reported among those patients who received each line of therapy. Costs for all patients are reported by line of therapy, but costs for those using specific treatment regimens are not provided for third- and fourth-line patients due to small sample sizes. ^a^Costs are in 2015/2016 euros and were inflated when necessary using the relevant inflation index [29]. ^b^Systemic treatment administration costs include the costs of administration, as well as the costs of outpatient visits or hospitalizations that were for chemotherapy administration. ^c^Treatment-related adverse event costs include costs associated with growth factors (which were assumed to have been used to prevent and/or treat neutropenia or anemia), as well as costs associated with hospitalizations to manage toxicities/side effects related to treatments or procedures. ^d^Costs are reported among those patients who received each line of therapy. ^e^Other direct health care costs include costs unrelated to anticancer therapy (i.e., other direct health care costs exclude costs associated with anticancer drug acquisition and administration and treatment-related adverse events). These other direct health care costs include costs associated with outpatient visits, accident and emergency visits, inpatient hospitalizations, inpatient long-term care and hospice care, and supportive care.

**Table 7:** Estimated health care costs related to treatment of advanced STS^a^ in France (*n*=201).

	First line^d^	Second line^d^	Third line^d^
*Systemic treatment costs*			
*N* (%)	201 (100)	108 (100)	46 (100)
Systemic drug and administration costs^b^			
** **Mean (SD)	€8,707.65 (€22,211.80)	€7,636.83 (€8,728.95)	€6,563.36 (€7,708.60)
** **Median	€4,381.76	€5,352.92	€3,431.74
** **Range (min, max)	(€355.42, €299,282.30)	(€710.84, €64,123.22)	(€710.84, €34,350.94)
Treatment-related adverse event costs^c^			
** **Mean (SD)	€1,644.20 (€2,645.60)	€1,224.50 (€2,127.10)	€916.60 (€1,729.00)
** **Median	€0.00	€0.00	€0.00
** **Range (min, max)	(€0.00, €15,656.30)	(€0.00, €9,273.60)	(€0.00, €5,833.00)
Total (all lines of therapy combined)			
** **Mean (SD)	€17,432.84 (€25,149.02)		
** **Median	€11,435.68		
** **Range (min, max)	(€355.42, €299,282.30)		
*Other direct health care costs* ^e^			
*N* (%)	201 (100)	108 (100)	46 (100)
Total cost			
** **Mean (SD)	€3,987.40 (€6,298.50)	€3,460.50 (€5,098.50)	€2,480.40 (€3,873.20)
** **Median	€1,553.60	€1,212.90	€547.10
** **Range (min, max)	(€0.00, €44,371.90)	(€0.00, €25,789.50)	(€0.00, €18,163.50)
Total cost (all available follow-ups)			
** **Mean (SD)	€6,934.77 (€8,066.56)		
** **Median	€4,142.27		
** **Range (min, max)	(€0.00, €44,451.04)		
Total cost, excluding surgery and radiotherapy			
** **Mean (SD)	€3,677.60 (€6,024.50)	€3,423.00 (€5,101.80)	€2,475.60 (€3,873.40)
** **Median	€1,431.50	€1,028.20	€547.10
** **Range (min, max)	(€0.00, €44,371.90)	(€0.00, €25,789.50)	(€0.00, €18,163.50)
Period of follow-up (weeks)			
** **Mean (SD)	25.80 (€22.60)	21.60 (16.50)	€19.40 (€13.60)
** **Median	20.10	18.20	€17.40
** **Range (min, max)	(0.30, €164.00)	(0.10, 115.10)	(€0.10, €55.00)
Weekly cost			
** **Mean (SD)	€1,156.50 (€8,298.50)	€827.40 (€3,863.60)	€1,001.80 (€4,570.30)
** **Median	€88.90	€88.20	€90.00
** **Range (min, max)	(€0.00, €103,534.50)	(€0.00, €36,894.00)	(€0.00, €30,693.20)
Weekly cost, excluding surgery and radiotherapy			
** **Mean (SD)	€1,039.60 (€7,929.10)	€823.90 (€3,864.10)	€1,001.40 (€4,570.40)
** **Median	€69.70	€81.00	€90.00
** **Range (min, max)	(€0.00, €103,534.50)	(€0.00, €36,894.00)	(€0.00, €30,693.20)

SD = standard deviation. Note: systemic treatment administration costs include the costs of administration and the costs of outpatient visits or hospitalizations that were for chemotherapy administration. Treatment-related adverse event costs include costs associated with growth factors (which were assumed to have been used to prevent and/or treat neutropenia or anemia) and costs associated with hospitalizations to manage toxicities/side effects related to treatments or procedures. For systemic treatment costs by line of therapy, costs are reported among those patients who received each line of therapy. Costs for all patients are reported by line of therapy, but costs for those using specific treatment regimens are not provided for third- and fourth-line patients due to small sample sizes. ^a^Costs are in 2015/2016 euros and were inflated when necessary using the relevant inflation index [30]. ^b^None of the systemic anticancer drugs reported required extra-DRG. Therefore, most drug costs in France are included within the administration costs and are not accounted for separately. The exception is pazopanib, for which a separate drug cost was applied. Systemic drug and administration costs include the cost of drug (pazopanib), costs of administration, as well as the costs of outpatient visits or hospitalizations that were for chemotherapy administration. ^c^Treatment-related adverse event costs include costs associated with growth factors (which were assumed to have been used to prevent and/or treat neutropenia or anemia), as well as costs associated with hospitalizations to manage toxicities/side effects related to treatments or procedures. ^d^Costs are reported among those patients who received each line of therapy. ^e^Other direct health care costs include costs unrelated to anticancer therapy (i.e., other direct health care costs exclude costs associated with anticancer drug acquisition and administration and treatment-related adverse events). These other direct health care costs include costs associated with outpatient visits, accident and emergency visits, inpatient hospitalizations, inpatient long-term care and hospice care, and supportive care.

## Data Availability

The data used to support the findings of this study are not publicly available but are available for collaborative research from Eli Lilly and Company, on reasonable request.

## Supplementary Materials

(Supplementary Materials) The supplementary appendix provides detailed information on unit costs, health care resource utilization, and hospitalization patterns by country.
